# The Prevalence of Histologically Confirmed Endometriosis in Salpingectomy Specimens for Hydrosalpinx and Subsequent Reproductive Outcomes

**DOI:** 10.1155/ogi/5574397

**Published:** 2026-10-14

**Authors:** Fatemeh Davari-Tanha, Sara Mehri, Raziye Akbari, Fatemeh Salehi, Razieh Mohammadkhani, Zeinab Sinaeifar, Arezu Mahin Behzadi

**Affiliations:** ^1^ Department of Obstetrics and Gynecology Yas Hospital Tehran University of Medical Sciences Tehran Iran tums.ac.ir; ^2^ Department of Obstetrics and Gynecology Imam Khomeini Hospital Tehran University of Medical Sciences Tehran Iran tums.ac.ir; ^3^ Department of Minimally Invasive Surgery Tehran University of Medical Sciences Tehran Iran tums.ac.ir; ^4^ Maternal Fetal and Neonatal Research Center Family Health Institute Tehran University of Medical Sciences Tehran Iran tums.ac.ir; ^5^ Valiasr Hospital Imam Khomeini Hospital Tehran University of Medical Sciences Tehran Iran tums.ac.ir

**Keywords:** endometriosis, histopathology, hydrosalpinx, infertility, salpingectomy

## Abstract

**Background:**

Hydrosalpinx, a fluid‐filled blocked fallopian tube, is a major cause of infertility and is mainly linked to pelvic inflammatory disease. However, its connection to endometriosis, especially hidden tubal endometriosis without visible signs, is not well studied. This research aimed to find the prevalence of confirmed endometriosis in salpingectomy samples removed due to hydrosalpinx and to evaluate the subsequent reproductive outcomes.

**Methods:**

A cross‐sectional study involved 70 infertile women undergoing laparoscopic salpingectomy at a specialized center. We gathered demographic, surgical, and pathological information from medical records. The main outcome was the occurrence of endometriosis based on histopathology. The secondary outcome was the rate of clinical pregnancies (either spontaneous or through ART) after salpingectomy. We used SPSS v23.0 for statistical analysis, considering *p* < 0.05 as significant.

**Results:**

The average age of participants was 37.38 ± 6.13 years. Clear histological evidence of endometriosis was found in 14.3% (10/70) of samples. Notably, 8% (4/50) of patients without a history of endometriosis had hidden tubal disease. The visual detection of endometriosis during laparoscopy was marginally significantly associated with a positive pathology (37.5% vs. 7.4%, *p* = 0.050). After salpingectomy, the total clinical pregnancy rate was 25.5% (12/47). However, none of the patients (0%) with a diagnosis of tubal endometriosis achieved pregnancy, while those with other pathologies had a 30% success rate.

**Conclusion:**

Examining salpingectomy samples is essential for detecting endometriosis. Women with histologically confirmed tubal endometriosis may represent a subgroup with potentially poorer reproductive outcomes. However, this observation is exploratory and requires validation in larger studies with adequate adjustment for age, ovarian reserve, and ART‐related factors.

## 1. Introduction

Infertility is a widespread health issue, affecting about 8% to 12% of couples of reproductive age around the world [1]. Tubal factor infertility is a major cause of female infertility, accounting for around 25% to 35% of cases. It often results from pelvic inflammatory disease (PID), surgical adhesions, and endometriosis [2, 3]. Among the various tubal problems, hydrosalpinx is a significant barrier to conception. This condition is characterized by a blocked tube and the buildup of serous fluid inside the tube [4].

Hydrosalpinx negatively impacts both natural and assisted reproductive success, and its effects are well known. Having a hydrosalpinx leads to a 50% decrease in implantation and pregnancy rates after in vitro fertilization (IVF) [5, 6]. The reasons for this severe effect are numerous. The fluid from a hydrosalpinx can harm embryos, hindering their development and causing apoptosis [7]. It can also wash away embryos after transfer and alter the expression of important biomarkers like integrins and HOXA10, creating an unfriendly environment in the uterus for implantation [8, 9]. As a result, the standard treatment is laparoscopic salpingectomy before IVF, which has been shown to double the chances of a clinical pregnancy and live birth [10, 11].

While PID is the most common cause of hydrosalpinx, endometriosis is another important, but often overlooked, factor. This chronic inflammatory condition involves the abnormal placement of tissue similar to endometrial tissue and affects about 10% of women. It contributes to nearly half of all infertility cases [12, 13]. In addition to creating pelvic adhesions and changing anatomy, endometriosis leads to ongoing pelvic inflammation. This inflammatory state can harm egg quality, sperm function, embryo development, and implantation [14, 15].

A critical but often neglected clinical situation is the presence of tubal endometriosis among patients undergoing surgery for hydrosalpinx. Endometriotic lesions can invade the tubal wall directly or cause scarring that blocks the tube, trapping fluids and forming a secondary hydrosalpinx [16, 17]. This can happen even without visible signs of pelvic endometriosis, making the diagnosis difficult without histological confirmation [18, 19]. This poses a significant challenge: a surgeon might mistakenly connect a hydrosalpinx to a past infection, overlooking the possible presence of tubal endometriosis. This distinction is important; it affects how patients are counseled, how all endometriotic lesions are surgically treated, and how chronic disease is managed in the long run.

Despite the clear treatment protocol of salpingectomy for hydrosalpinx to improve IVF results, there is a noticeable lack of research. Few studies have looked at the percentage of hydrosalpinges that show direct evidence of tubal endometriosis upon examination. Additionally, while IVF outcomes after salpingectomy are documented, there is little information on later natural conception rates in this group. Knowing how many in this population actually have endometriosis is vital for proper disease diagnosis, effective surgical care, and accurate patient prognosis.

This study aims to fill these important gaps. Our main goal is to find how common histologically confirmed endometriosis is in the fallopian tubes removed due to a preoperative diagnosis of hydrosalpinx in infertile individuals. Our secondary goal is to assess the rate of successful pregnancy, whether achieved naturally or through assisted reproductive technology (ART), following salpingectomy. Our study design and sample size offer enough predictive power for the first goal of measuring prevalence. However, the outcome of pregnancy rates might be limited. While our study is adequately powered to estimate the prevalence of tubal endometriosis, the analysis of reproductive outcomes is exploratory due to the limited number of pregnancy events and should be interpreted with caution.

## 2. Materials and Methods

### 2.1. Study Design and Ethical Approval

This retrospective observational cohort study took place at a tertiary university–based hospital. Since data collection was retrospective, the ethics committee waived the requirement for informed consent. All patients completed the informed consent form and their data remained anonymous and strictly confidential.

### 2.2. Study Population and Sampling

The study involved women of reproductive age who presented with infertility and underwent laparoscopic unilateral or bilateral salpingectomy due to an intraoperative finding of hydrosalpinx from September 2021 to September 2024.

A convenience (nonprobability) sampling method was used. Based on an estimated prevalence of 10% for pathological endometriosis in fallopian tubes (from previous studies [1]), a sample size of 68 was needed to estimate the proportion with a 95% confidence level and a 7% margin of error. The calculation formula was:

*n* = (*Z* ^2^∗*P*(1 − *P*))/*d* ^2^, where *Z* = 1.96 (for 95% CI), *p* = 0.1, and *d* = 0.07. To cover possible missing data, a total of 70 patients were included.

Inclusion criteria included women aged 18–43 years who were diagnosed with infertility, had laparoscopic salpingectomy (unilateral or bilateral), and had hydrosalpinx confirmed visually during the laparoscopic procedure.

Exclusion criteria consisted of patients who refused salpingectomy, those without a documented history of infertility, and cases where hydrosalpinx was not found during surgery.

### 2.3. Data Collection and Variables

Data were collected retrospectively from patients’ medical records, surgical notes, and pathology reports using a structured data collection sheet created by the researchers. A pilot test checked the accuracy on 10 random records before the complete data extraction began.

The collected data included

- Demographic and baseline characteristics: Age, body mass index (BMI), type of infertility (primary vs. secondary), history of abortion, and history of abdominal/pelvic surgeries (laparotomy, laparoscopy, or both).
- Presence of underlying medical conditions (e.g., diabetes, hypertension, hypothyroidism).
- Detailed information from the surgical reports, such as the laterality of hydrosalpinx (unilateral or bilateral), presence of endometriosis (e.g., deep infiltrating endometriosis [DIE], kissing ovaries, involvement of uterosacral ligaments, bowel, or ureter), tubal obstruction (unilateral or bilateral), fimbriosis, tortuous tubes, and reduced sliding.

The final histopathological report of the excised fallopian tube(s) served as the primary outcome measure. Reports were classified as

1. No specific pathological change,
2. Mild hydrosalpinx,
3. Paratubal cyst,
4. Endometriosis (presence of endometrial glands and stroma), or
5. Congestion.

Postoperative reproductive outcomes were recorded based on whether conception was attempted spontaneously or through ART. Due to the retrospective nature of the study and incomplete availability of detailed ART‐related parameters (including stimulation protocols, embryo characteristics, and number of transferred embryos), ART‐associated and spontaneous pregnancies were analyzed together. The follow‐up period was defined as the interval between salpingectomy and the last recorded fertility assessment or documented pregnancy outcome. The mean follow‐up duration was [mean ± SD: 27.3 ± 4.2] months (range: 18–36 months), extracted from available medical records. Variability in follow‐up duration was considered when interpreting reproductive outcomes, and patients were followed until the date of last documented contact or until pregnancy was achieved, whichever occurred first.

### 2.4. Statistical Analysis

All statistical analyses were carried out using IBM SPSS Statistics for Windows, Version 23.0 (Armonk, NY: IBM Corp). Descriptive statistics were shown as mean ± standard deviation (SD) for continuous variables (e.g., age, BMI, duration of infertility) and as frequency (percentage) for categorical variables. The primary outcome, the prevalence of histologically confirmed endometriosis, was reported as a percentage with a 95% confidence interval (CI). To examine associations between categorical variables (e.g., history of endometriosis and pathological findings, type of infertility and pregnancy success), Chi‐square (*χ* ^2^) or Fisher’s exact test was used as appropriate. An independent samples *t*‐test compared the means of continuous variables between groups. A *p*‐value of less than 0.05 was considered statistically significant for all tests. Multivariable logistic regression analysis was considered to evaluate independent predictors of clinical pregnancy. However, because the number of pregnancy events was limited (*n* = 12 overall) and the tubal endometriosis subgroup was small, multivariable modeling was not performed to avoid model instability and overfitting. Important potential confounders that could not be adjusted for in the analysis included ovarian reserve markers (anti‐Müllerian hormone [AMH] levels and antral follicle count), details of ART protocols (stimulation regimens, embryo quality, and number of transferred embryos), and duration of follow‐up, all of which may independently influence reproductive outcomes and limit the interpretability of the observed associations between tubal endometriosis and pregnancy rates. The absence of multivariable adjustment means that the reported pregnancy outcomes should be considered exploratory and hypothesis‐generating rather than definitive. A formal survival analysis (e.g., Kaplan–Meier) was not performed because precise dates of conception attempts were not uniformly recorded for all patients.

## 3. Results

A total of 70 women who met the inclusion criteria and had laparoscopic salpingectomy for hydrosalpinx between September 2021 and September 2024 were included in the final analysis. The demographic and clinical profile of the group is shown in Table 1.

**TABLE 1:** Baseline demographic and clinical characteristics of the study population (*N* = 70).

Characteristic	Category	Value/*n*	Percentage (%)
Age (years)	Mean ± SD	37.38 ± 6.13	—
BMI (kg/m^2^)	Mean ± SD	26.39 ± 3.86	—
Duration of infertility (years)	Mean ± SD	6.18 ± 5.48	—
Type of infertility	Primary	42	60.0
Secondary	28	40.0	—
History of adverse pregnancy outcomes (in secondary infertility group, *n* = 28)^∗^	Ectopic pregnancy	8	28.6
Miscarriage	3	10.7	—
Both^∗∗^	1	3.6	—
Live births only	6	21.4	—
Previous abdominal surgery	Yes	29	41.4
No	41	58.6	—
Type of previous surgery	Laparotomy	16	22.9
Laparoscopy	9	12.9	—
Both	4	5.7	—
Comorbidities	Any comorbidity	17	24.3
Hypothyroidism	7	10.0	—
Hypertension	4	5.7	—
Diabetes mellitus	3	4.3	—

^∗^Percentages are calculated among patients with secondary infertility (*n* = 28).

^∗∗^Both = history of both ectopic pregnancy and miscarriage.

The average age of the study participants was 37.38 ± 6.13 years. Because maternal age is an important determinant of reproductive outcome, patients were additionally categorized according to age. A considerable proportion of participants were aged ≥ 35 years, reflecting an advanced reproductive age group that may influence pregnancy outcomes independently of tubal pathology. The average BMI was 26.39 ± 3.86 kg/m^2^, indicating that most patients were in the overweight range according to WHO classification. The infertility burden was significant, with a mean duration of 6.18 ± 5.48 years, highlighting their long‐standing fertility struggles.

Most of the patients had primary infertility, affecting 60% (*n* = 42), while 40% (*n* = 28) had secondary infertility. Among the patients with secondary infertility, a detailed history of previous pregnancies revealed that eight patients had a history of ectopic pregnancy, three patients had a history of miscarriage, one patient had a history of both one ectopic pregnancy and two miscarriages, and the remaining six patients (21.4% of the secondary infertility group) had all previous pregnancies resulting in live births.

A notable portion of patients (41.4%, *n* = 29) had undergone previous abdominal or pelvic surgery, which may contribute to tubal issues. Among these, laparotomy was the most common procedure (*n* = 16), followed by laparoscopy (*n* = 9), and a combination of both (*n* = 4). Comorbid conditions were present in 24.3% (*n* = 17) of the group. The most common endocrine disorder was hypothyroidism (10%, *n* = 7), followed by hypertension (5.7%, *n* = 4) and diabetes mellitus (4.3%, *n* = 3). Uterine Müllerian anomalies, which may be linked to infertility, were found in 15.7% (*n* = 11) of the patients.

Detailed intraoperative findings offered important insights into the pelvic issues linked to hydrosalpinx. The side affected by hydrosalpinx was a significant surgical observation. Bilateral hydrosalpinx occurred much more often, affecting 62.9% (*n* = 44) of patients, while unilateral involvement was seen in 37.1% (*n* = 26) of cases.

In addition to the main reason for surgery, a variety of other pelvic issues were documented during laparoscopy (Table 2). Many of these conditions were first noticed or suspected during preoperative ultrasonography. Tubal obstruction was a common finding, with bilateral obstruction seen in 35.7% (*n* = 25) of patients and unilateral obstruction in 22.9% (*n* = 16). Signs of DIE were visually noted in 11.4% (*n* = 8) of cases. Involvement of the uterosacral ligaments was observed in 14.3% of patients (bilateral: 12.9%, *n* = 9; unilateral: 1.4%, *n* = 1). Bowel involvement occurred in 11.4% (*n* = 8) of patients, while ureteric involvement was less frequent at 4.3% (*n* = 3). Other important findings included “kissing ovaries” (2.9%, *n* = 2), reduced tubal sliding (4.3%, *n* = 3), and tortuous tubes (8.6%, *n* = 6). Notably, clear visual signs of endometriosis (such as powder‐burn lesions and endometriomas) during laparoscopy were reported in only 22.9% (*n* = 16) of the entire group.

**TABLE 2:** Intraoperative laparoscopic findings (*N* = 70).

Laparoscopic finding	*n*	%
Hydrosalpinx laterality		
Unilateral	26	37.1
Bilateral	44	62.9
Tubal obstruction		
Unilateral	16	22.9
Bilateral	25	35.7
Deep infiltrating endometriosis (DIE)	8	11.4
Uterosacral ligament involvement		
Unilateral	1	1.4
Bilateral	9	12.9
Bowel involvement	8	11.4
Ureteric involvement	3	4.3
Kissing ovaries	2	2.9
Tortuous tube(s)	6	8.6
Reduced sliding	3	4.3
Obvious visual endometriosis	16	22.9

The histopathological analysis of the excised fallopian tubes was the main focus of this study and provided a clear diagnosis. The results are summarized in Table 3.

**TABLE 3:** Final histopathological diagnosis of excised fallopian tubes (*N* = 70).

Pathological diagnosis	*n*	%	95% Confidence interval
No specific pathological change	36	51.4%	39.7%–63.0%
Mild hydrosalpinx	13	18.6%	10.9%–29.4%
Paratubal cyst	8	11.4%	5.6%–21.1%
Endometriosis	10	14.3%	7.5%–24.9%
Congestion	3	4.3%	1.2%–12.4%
Total	70	100.0%	—

The most common outcome was the absence of specific pathological changes, which occurred in 51.4% (*n* = 36) of the specimens. This indicates that the hydrosalpinx in these cases was likely due to nonspecific salpingitis or aftereffects of an infection, without active or specific histologic features. The second most frequent finding was mild hydrosalpinx, seen in 18.6% (*n* = 13) of the cases, followed by paratubal cysts in 11.4% (*n* = 8).

The main finding of this research was that clear histological evidence of endometriosis, shown by the presence of endometrial glands and stroma, was found in 14.3% (*n* = 10) of all fallopian tubes removed for a preoperative and intraoperative diagnosis of hydrosalpinx. This represents a notable proportion of cases with histologically confirmed tubal endometriosis among patients undergoing salpingectomy for hydrosalpinx. A small number of specimens showed vascular congestion, at 4.3% (*n* = 3).

Further analysis was conducted to explore factors associated with a pathological diagnosis of endometriosis (Table 4):

**TABLE 4:** Association between patient history and pathological diagnosis of endometriosis.

Factor	Pathology: Endometriosis	Pathology: Other	*p*‐value
History of endometriosis			0.028
Yes (*n* = 20)	6 (30.0%)	14 (70.0%)	
No (*n* = 50)	4 (8.0%)	46 (92.0%)	
Visual endometriosis at surgery			0.050
Yes (*n* = 16)	6 (37.5%)	10 (62.5%)	
No (*n* = 54)	4 (7.4%)	50 (92.6%)	
Hydrosalpinx laterality			0.624
Unilateral (*n* = 26)	2 (7.7%)	24 (92.3%)	
Bilateral (*n* = 44)	8 (18.2%)	36 (81.8%)	
Age group			0.030
≤ 30 years (*n* = 12)	0 (0.0%)	12 (100.0%)	
> 30 years (*n* = 58)	10 (17.2%)	48 (82.8%)	
BMI category			0.880
< 30 kg/m^2^ (*n* = 55)	7 (12.7%)	48 (87.3%)	
≥ 30 kg/m^2^ (*n* = 15)	3 (20.0%)	12 (80.0%)	

A significant association was found between a patient’s history of endometriosis and the presence of tubal endometriosis on pathology (*p* = 0.028). Among the 20 patients with a clinical history of endometriosis, 30% (*n* = 6) had their diagnosis confirmed in the tubal specimen. Most notably, occult tubal endometriosis was found in 8% (*n* = 4) of the 50 patients without a prior history of the disease. This highlights a group that would have been misclassified without histological examination.

The presence of noticeable endometriosis during laparoscopy strongly predicted a positive pathological result (*p* = 0.050). In patients where the surgeon observed visual signs of endometriosis, 37.5% (6 out of 16) had endometriosis confirmed on tubal pathology. In comparison, only 7.4% (4 out of 54) of patients without visual signs had occult tubal endometriosis.

The occurrence of endometriosis was higher in cases of bilateral hydrosalpinx (18.2%, 8/44) than in unilateral hydrosalpinx (7.7%, 2/26), although this difference was not statistically significant (*p* = 0.624).

No cases of tubal endometriosis were found in women aged 30 years or younger (0/12). The prevalence rose to 17.2% (10/58) in women over 30. Cases were observed in the following age groups: 31–35 (18.8%), 36–40 (18.2%), and 41–45 (18.8%). This difference was statistically significant (*p* = 0.030).

No significant connections were found between the pathological diagnosis of endometriosis and BMI (*p* = 0.880), type of infertility (primary vs. secondary, *p* = 0.267), or the presence of Müllerian anomalies (*p* = 0.592).

Postoperative follow‐up focused on the important outcome of achieving pregnancy. After salpingectomy, 67.1% (*n* = 47) of patients actively tried to conceive, either naturally or through ART. The overall clinical pregnancy rate among these women was 25.5% (12 out of 47). Among the 47 patients who attempted conception after salpingectomy, pregnancy outcomes were further stratified according to histological diagnosis. Twelve patients achieved clinical pregnancy, all of whom belonged to the group without histologically confirmed tubal endometriosis. Among patients without tubal endometriosis, 12/40 (30.0%) achieved pregnancy, while 28/40 (70.0%) did not. In contrast, none of the patients with histologically confirmed tubal endometriosis achieved pregnancy during the available follow‐up period (0/7, 0%). Although the difference in pregnancy rates (30.0% vs. 0%) was clinically noteworthy, statistical comparison was limited by the small number of patients in the endometriosis subgroup. The observed association did not reach statistical significance (*p* = 0.192), and these findings should be interpreted cautiously. Further analysis included the 47 patients who attempted conception (Table 5). In response to the clinically relevant concern regarding advancing maternal age, we stratified pregnancy outcomes by age groups (Table 6). Women aged ≤ 30 years had a significantly higher clinical pregnancy rate compared to those aged > 30 years (41.7% vs. 20.0%, *p* = 0.045), confirming the well‐established negative impact of age on fertility outcomes. Notably, the prevalence of histologically confirmed tubal endometriosis was consistently elevated across all age subgroups above 30 years (31–35: 18.8%, 36–40: 18.2%, and 41–45: 18.8%), suggesting that the burden of endometriosis increases with age and may interact with age‐related decline in reproductive potential. While a specific stratification at the exact 35‐year threshold was not feasible due to the limited sample size and retrospective design, the significant age effect observed at the 30‐year cut‐off adequately addresses the reviewer’s concern regarding age as a major confounding factor in pregnancy outcomes.

**TABLE 5:** Pregnancy outcomes based on patient subgroups (among *n* = 47 who attempted conception).

Subgroup	Attempted pregnancy (*n*)	Clinical pregnancy achieved, *n* (%)	*p*‐value
Overall	47	12 (25.5%)	—
Pathology diagnosis			0.192
Endometriosis	7	0 (0.0%)	
Other diagnoses	40	12 (30.0%)	
Type of infertility			0.686
Primary	28	7 (25.0%)	
Secondary	19	5 (26.3%)	
Age group			0.045
≤ 30 years	12	5 (41.7%)	
> 30 years	35	7 (20.0%)	
Müllerian anomaly			0.678

**TABLE 6:** Age‐stratified pregnancy outcomes among patients who attempted conception (*n* = 47).

Age group	Attempted conception (*n*)	Clinical pregnancy achieved, *n* (%)	*p*‐value
≤ 30 years	12	5 (41.7%)	0.045
> 30 years	35	7 (20.0%)	

## 4. Discussion

This study offers important insights into the causes of hydrosalpinx in people dealing with infertility and the reproductive outcomes after corrective surgery. Our main finding—that 14.3% of fallopian tubes removed due to hydrosalpinx showed clear signs of endometriosis—highlights a significant issue that might often go unnoticed. This rate of hidden tubal endometriosis, especially the 8% found in women with no previous history of the disease, points to a diagnostic gap where the presence of tubal endometriosis may be overlooked when evaluating patients with hydrosalpinx.

The rates we report match existing literature but also provide new information. Our findings support the case report by Ozturk et al. (2010), which first pointed out that intraluminal tubal endometriosis can mimic hydrosalpinx [18]. It also backs up a larger study by Jiao et al. (2022), which showed that tubal endometriosis is often found during laparoscopy for infertility [16]. However, our study adds an important detail by quantifying this prevalence within a series of patients who underwent salpingectomy specifically for hydrosalpinx, giving a clearer picture of the impact of endometriosis in this surgical group.

The strong statistical connections we found further support the importance of our results. The significant link between a patient’s history of endometriosis and positive tubal pathology (*p* = 0.028) makes sense and confirms that the disease can appear in the tubal lumen. This histological confirmation is important because diagnosing can be difficult. Pathologists may sometimes mistake endometrial stroma for something else. In our study, we carefully checked all specimens for both endometrial glands and stroma to avoid this common mistake. More importantly, the significant relationship with visual signs of endometriosis during laparoscopy (*p* = 0.050) shows that surgeons are often right to suspect the disease. However, it is critical to note that 7.4% of patients without visible signs of endometriosis still had hidden tubal disease. This finding calls for a review of diagnostic practices, suggesting that histological analysis of salpingectomy samples should become standard. However, our study adds a crucial layer by quantifying this prevalence within a consecutive series of patients undergoing salpingectomy specifically for hydrosalpinx, providing a clearer estimate of the burden of endometriotic etiology in this specific surgical cohort.

Another noteworthy finding was the complete absence of tubal endometriosis in women aged 30 or younger (0/12), with all cases occurring in women older than 30. This suggests that tubal endometriosis might appear later in life, possibly due to the ongoing nature of the disease. Over time, repeated inflammation and minor bleeding from endometrial implants near the tube could cause fibrosis, scarring, and ultimately lead to fimbrial occlusion and hydrosalpinx. This age‐related trend supports the view of endometriosis as a disease that changes over time [12, 20]. The ongoing inflammation associated with endometriosis, involving prostaglandins, cytokines, and metalloproteinases, can lead to continued tubal inflammation, fibrosis, and eventually fimbrial blockage [18]. This matches the findings from Xue et al. (2020), who observed the progressive and often uneven nature of tubal endometriosis [17].

The assessment of reproductive outcomes following salpingectomy produced significant, though concerning, results. The overall clinical pregnancy rate of 25.5% shows the clear benefit of salpingectomy in removing toxic hydrosalpinx fluid and creating a better environment for implantation, a well‐established effect shown in various studies [10, 11]. However, the significant difference in outcomes between pathological groups is the most striking aspect of our study: none of the patients (0%) with histologically confirmed tubal endometriosis achieved a clinical pregnancy, compared to a 30% success rate in those with other pathologies. This serious gap in pregnancy outcomes stems from the complex nature of endometriosis. One major factor is the common occurrence of endometriosis alongside adenomyosis, where endometrial‐like tissue grows into the uterine muscle wall [19, 21–24]. This link is well‐known, and adenomyosis may limit successful implantation or rates of pregnancy [25, 26]. The poorer reproductive outcomes observed in this subgroup may be related to the broader impact of endometriosis on reproductive function, including possible effects on pelvic anatomy, ovarian reserve, and implantation capacity [27, 28]. So, even after surgically fixing the mechanical issue of hydrosalpinx, these underlying problems still create serious obstacles to achieving a successful pregnancy.

This major difference, while not statistically significant due to the small size of the endometriosis group, is still clinically important. However, these findings should be interpreted with caution because the number of patients with histologically confirmed tubal endometriosis who attempted conception was limited (*n* = 7). The small subgroup size substantially reduces statistical power and increases the possibility that the observed difference may be influenced by random variation. Therefore, although the absence of pregnancy in this subgroup is clinically noteworthy, it should not be considered definitive evidence of treatment failure without confirmation in larger prospective studies. When tubal endometriosis is identified in patients undergoing salpingectomy for hydrosalpinx, it indicates a more severe disease state. The failure to achieve pregnancy after salpingectomy in this subgroup implies that fertility issues go beyond just the mechanical problem of hydrosalpinx. These women likely face more complex problems related to endometriosis. The poorer reproductive outcomes observed in patients with histologically confirmed tubal endometriosis may reflect the multifactorial effects of endometriosis on fertility, including possible alterations in pelvic environment, implantation capacity, and ovarian function. However, these factors could not be fully evaluated in the present retrospective cohort [8, 9, 14, 15, 29].

For patients with hydrosalpinx and histologically confirmed tubal endometriosis, salpingectomy alone may not be sufficient to address all factors affecting reproductive outcomes. Our findings show that a more thorough management approach is necessary. A complete preoperative evaluation is vital for proper diagnosis. When tubal endometriosis or other visible endometriotic lesions are identified, appropriate management should be considered according to the extent of disease. Additionally, we recommend a planned approach to preserving fertility. We usually offer embryo freezing before extensive endometriosis surgery. This method keeps fertility options open while allowing for the chance of natural conception afterward if circumstances are right. If a natural pregnancy does not happen, the frozen embryos provide a secure option through Frozen Embryo Transfer (FET) [30, 31]. In situations where viable eggs cannot be obtained from the patient before surgery, or when severe adenomyosis or intrauterine adhesions affect the uterus’s ability to support pregnancy, considering alternatives like egg donation or gestational surrogacy may be necessary.

This study has several limitations. First, the retrospective design may have introduced selection bias and information bias due to dependence on available medical records, and the absence of standardized follow‐up protocols may have contributed to variability in outcome ascertainment. Second, although the sample size was adequate for estimating the prevalence of histologically confirmed tubal endometriosis, it limited the statistical power of subgroup analyses, particularly pregnancy outcomes among patients with tubal endometriosis. The small number of patients with tubal endometriosis who attempted conception (*n* = 7) precluded meaningful statistical comparisons and multivariable adjustment, meaning the observed 0% pregnancy rate in this subgroup cannot be considered definitive evidence of treatment failure. Third, ART‐related factors were not fully stratified because detailed treatment parameters (including stimulation protocols, embryo quality, and number of transferred embryos) were not consistently available, and this may have influenced pregnancy outcomes. The duration of follow‐up also varied between patients, and information on whether patients continued trying to conceive after the follow‐up period was incomplete, potentially underestimating pregnancy rates. Finally, important reproductive confounders, including ovarian reserve markers such as AMH levels and antral follicle count, were not available for all patients and could not be incorporated into the analysis. Consequently, the independent contribution of tubal endometriosis to reproductive outcomes could not be fully separated from the effects of advancing maternal age, diminished ovarian reserve, or underlying infertility factors.

## 5. Conclusion

In summary, our findings suggest a possible link between tubal endometriosis and hydrosalpinx formation. Women with hydrosalpinx and histologically confirmed tubal endometriosis might represent a distinct subgroup with a potentially worse reproductive outlook. However, this is an exploratory finding that requires validation in larger cohort studies before any definitive conclusions can be drawn.

## Funding

No funding was received for this manuscript.

## Conflicts of Interest

The authors declare no conflicts of interest.

## Data Availability

The data that support the findings of this study are available from the corresponding author upon reasonable request.
